# Volatile profiling reveals intracellular metabolic changes in *Aspergillus parasiticus*: *veA *regulates branched chain amino acid and ethanol metabolism

**DOI:** 10.1186/1471-2091-11-33

**Published:** 2010-08-24

**Authors:** Ludmila V Roze, Anindya Chanda, Maris Laivenieks, Randolph M Beaudry, Katherine A Artymovich, Anna V Koptina, Deena W Awad, Dina Valeeva, Arthur D Jones, John E Linz

**Affiliations:** 1 Department of Food Science and Human Nutrition, Michigan State University, East Lansing, MI, USA; 2 Department of Microbiology and Molecular Genetics, Michigan State University, East Lansing, MI, USA; 3 Department of Horticulture, Michigan State University, East Lansing, MI, USA; 4 Department of Biochemistry and Molecular Biology, Michigan State University, East Lansing, MI, USA; 5 Department of Chemistry, Michigan State University, East Lansing, MI, USA; 6 National Food Safety and Toxicology Center, Michigan State University, East Lansing, MI, USA

## Abstract

**Background:**

Filamentous fungi in the genus *Aspergillus *produce a variety of natural products, including aflatoxin, the most potent naturally occurring carcinogen known. Aflatoxin biosynthesis, one of the most highly characterized secondary metabolic pathways, offers a model system to study secondary metabolism in eukaryotes. To control or customize biosynthesis of natural products we must understand how secondary metabolism integrates into the overall cellular metabolic network. By applying a metabolomics approach we analyzed volatile compounds synthesized by *Aspergillus parasiticus *in an attempt to define the association of secondary metabolism with other metabolic and cellular processes.

**Results:**

Volatile compounds were examined using solid phase microextraction - gas chromatography/mass spectrometry. In the wild type strain *Aspergillus parasiticus *SU-1, the largest group of volatiles included compounds derived from catabolism of branched chain amino acids (leucine, isoleucine, and valine); we also identified alcohols, esters, aldehydes, and lipid-derived volatiles. The number and quantity of the volatiles produced depended on media composition, time of incubation, and light-dark status. A block in aflatoxin biosynthesis or disruption of the global regulator *veA *affected the volatile profile. In addition to its multiple functions in secondary metabolism and development, VeA negatively regulated catabolism of branched chain amino acids and synthesis of ethanol at the transcriptional level thus playing a role in controlling carbon flow within the cell. Finally, we demonstrated that volatiles generated by a *veA *disruption mutant are part of the complex regulatory machinery that mediates the effects of VeA on asexual conidiation and sclerotia formation.

**Conclusions:**

1) Volatile profiling provides a rapid, effective, and powerful approach to identify changes in intracellular metabolic networks in filamentous fungi. 2) VeA coordinates the biosynthesis of secondary metabolites with catabolism of branched chain amino acids, alcohol biosynthesis, and β-oxidation of fatty acids. 3) Intracellular chemical development in *A. parasiticus *is linked to morphological development. 4) Understanding carbon flow through secondary metabolic pathways and catabolism of branched chain amino acids is essential for controlling and customizing production of natural products.

## Background

Secondary metabolites are low-molecular-weight natural products generated by filamentous fungi, plants, algae, bacteria, and animals in response to environmental abiotic and biotic stimuli. Secondary metabolites have a strong impact on humankind via their application in health, medicine, agriculture, and industry; they include useful (e.g. antibiotics) and detrimental compounds (e.g. mycotoxins).

Filamentous fungi produce a broad range of secondary metabolites. Each fungal species can synthesize multiple secondary metabolites, and these metabolites vary from species to species as well thus enabling the use of secondary metabolite profiling in the chemotaxonomy of filamentous fungi [1-3]. The complex network of secondary metabolism is connected to basic (primary) metabolism. Secondary metabolites are derived from compounds formed during primary metabolism, e.g. amino acids, nucleotides, carbohydrates, acyl-CoA (reviewed in [4]). Each secondary metabolic pathway accomplishes its specific function (although often unknown) as part of cellular metabolism and appears to provide "active safety" mechanisms for the producer enhancing survival in the continuously changing environment [5-10].

*Aspergillus spp*. produce an array of secondary metabolites including aflatoxin, cyclopiazonic acid, aflatrem, patulin, penicillin, kojic acid, lovastatin, carotenoids, and spore pigments; novel secondary metabolites have also been discovered that are synthesized from so called silent gene clusters in *A. nidulans*, such as terrequinone A, monodictyphenone, emodins, and polyketides [11]. Fungal-bacterial physical interactions have been shown to induce silent secondary metabolic gene cluster expression in *A. nidulans *required for biosynthesis of the polyketide orsellinic acid [5,9].

Aflatoxin biosynthesis is one of the most highly characterized secondary metabolic pathways [12-15]. In contrast, pathways for the synthesis of many other secondary metabolites, e.g. patulin, cyclopiazonic acid, aflatrem, and kojic acid, are poorly understood [16-18]. Molecular regulation of aflatoxin biosynthesis is complex and involves control of gene expression at the level of the individual gene and at the level of the entire gene cluster [14,15,19]. Biosynthesis of aflatoxin initiates during a transition from exponential growth to stationary phase, and closely correlates with fungal development (conidiospore, cleistothecia, and sclerotia formation) [20-22]. AflR, a positive aflatoxin pathway regulator, is a transcription factor that controls at least in part expression of several genes in the aflatoxin gene cluster [23]. VeA, a global regulator of secondary metabolism, links response to light with secondary metabolism and fungal development; this response is mediated through formation of a protein complex VelB/VeA/LaeA [24]. LaeA is a nuclear methyl transferase that through protein-protein interactions mediates regulation of secondary metabolism and development [21]. Aflatoxin biosynthesis is precisely orchestrated within the cell; the early reactions are reported to occur in peroxisomes [25]; recent evidence from our laboratory suggests that specific early steps as well the middle and late steps are carried out in specialized trafficking vesicles, called aflatoxisomes, which are also involved in export of the toxin outside the cell [12]. A novel role for VeA in coordination of aflatoxisome development with aflatoxin biosynthesis was recently discovered [4,12]. Biosynthesis of aflatoxins appears to fulfill multiple biochemical and biological functions including removal of acetate, protection of the genome from UV damage [26], quenching oxidative stress [27-29], protection from insects [30,31], and regulation of conidiation, and sclerotia development [22,32-34].

In order to manipulate efficiently secondary metabolism (to enhance production of beneficial metabolites and to control production of detrimental ones) we must understand the "molecular switch" mechanism that controls the initiation of secondary metabolism. Reaching this understanding requires a cooperative effort from genomic, proteomic, and metabolomic research. Despite advances in knowledge about the genes involved in biosynthesis and the regulation of many secondary metabolitic pathways, a detailed understanding of how secondary metabolism integrates with other metabolic and cellular processes is still not available [12,25,35-39].

Metabolomics is a powerful tool to characterize the metabolic state of the cell and to discover new metabolites and biochemical pathways [40]. Volatiles, one important group of cellular metabolites, represent a significant portion of the metabolome. Many organic compounds are present in the volatile phase including acids, alcohols, aldehydes, esters, short chain fatty acids, lipid oxides, terpenes, and phenolics. In this study we applied volatile profiling analysis for gaining rapid access to information on intracellular metabolism in the fungus. Specifically, we examined carbon flow in the presence or absence of secondary metabolism in *A. parasiticus *using wild type and mutant strains carrying genetic defects specifically in aflatoxin biosynthesis and in VeA, a global regulator of secondary metabolism. The volatile metabolites generated by the fungus were analyzed using solid phase microextraction - gas chromatography/mass spectrometry (SPME-GC/MS). This analytical approach is a non-invasive and solvent-free absorption technique that is used in analysis of volatile compounds from the headspace above the sample [41]; the technique has been widely employed in volatile analysis (profiling) of plants, yeast, and bacteria because it is accurate, sensitive, and robust [41-48]. To conduct this procedure, the outer polymer coating of a fused silica fiber absorbs volatiles from the headspace in the growth environment; the volatiles are then desorbed in the hot GC inlet and chromotographed in the usual manner. The separated compounds are subsequently identified by mass spectrometry.

Using SPME-GC/MS volatile profiling analysis we demonstrated that a genetic block in aflatoxin biosynthesis or disruption of the global regulator *veA *re-directs intracellular carbon flow. Specifically, we observed that VeA negatively regulates catabolism of branched chain amino acids and the synthesis of ethanol in *A. parasiticus*; these metabolic changes were mediated at least in part at the transcriptional level. We also showed that volatile metabolites generated under the control of VeA may participate in the molecular machinery that regulates conidiation and sclerotia formation.

## Results

### Profiling of volatile compounds in *A. parasiticus *SU-1

We withdrew samples from cultures at regular intervals during growth and analyzed volatiles following a 1 to 2 h equilibration period; this sampling method resulted in stable and reproducible measurements. We also analyzed volatile compounds in a control injection that originated from the SPME fiber, the glass vial, and the screw cap and valve; these volatiles were excluded from the analysis of volatiles detected in the fungal culture headspace. The relative amounts of volatiles produced in culture were assessed based on instrument response [42]; these compounds were designated as possible, or putative, fungal metabolites since they could be identified by comparison with a mass spectrum library. Since the *A. parasiticus *strains used in the study (Table 1) did not differ significantly in growth rate in liquid YES medium (Additional File 1, Figure S1), the relative intensity change of all masses detected was also related to the levels of the compounds produced in culture. Compounds with no match in the NIST mass spectrum library were defined as unknown. Ethanol levels produced by the fungus in culture were compared to standards. Thus, all volatiles detected fell into one of three categories: 1) known compounds identified with standards (ethanol); 2) putative compounds identified by a match in NIST mass spectrum library; and 3) unknown compounds.

**Table 1:** Strains used in the study

Strain	Genotype	Source
*A. parasiticus *SU-1 (ATCC 56775)	wild type	ATCC
*A. parasiticus *ATCC 36537	*ver-1 wh *	ATCC
*A. parasiticus *Δ*ve*A (TJW35.21)	*ver-1 wh pyrG^- ^ΔveA::pyrG *	Calvo *et al*., 2004 [31]
*A. parasiticus *AFS10 (ATCC 24690)	*aflR*	Cary *et al*., 2002 [32]
*A. parasiticus *B62	*niaD nor-1 br-1 *	ATCC
*A. nidulans *FGSC4	wild type	FGSC

**Figure 1:**
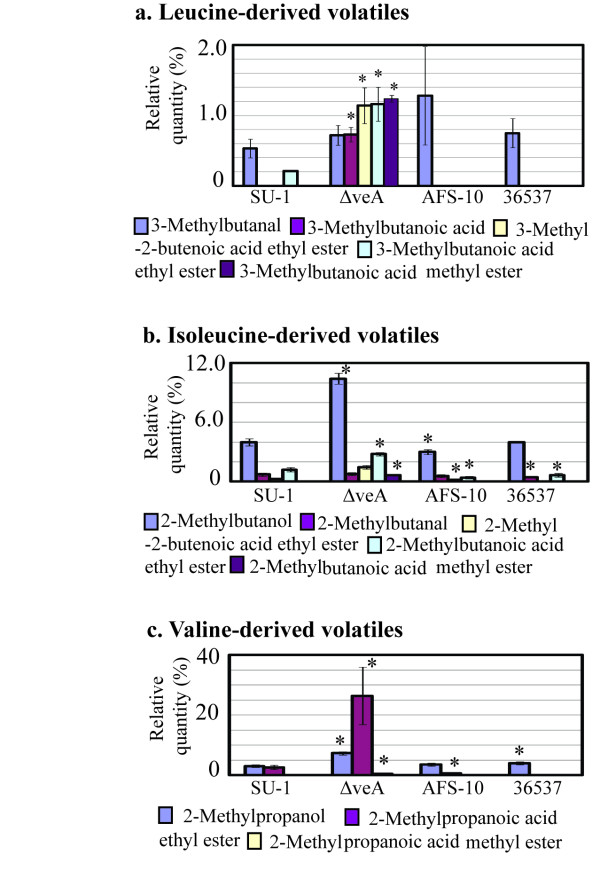
**Branched chain amino acid-derived volatiles generated by *A. parasiticus *strains grown in YES for 72 h in the dark**. Conidiospores were inoculated into 100 ml of liquid YES medium at 10^4^/ml and the cultures were grown at 30°C, with shaking at150 rpm, in the dark for 72 h. Volatiles were analyzed as described in Methods. *, statistically significant difference as compared with SU-1, P < 0.01.

**Figure 2:**
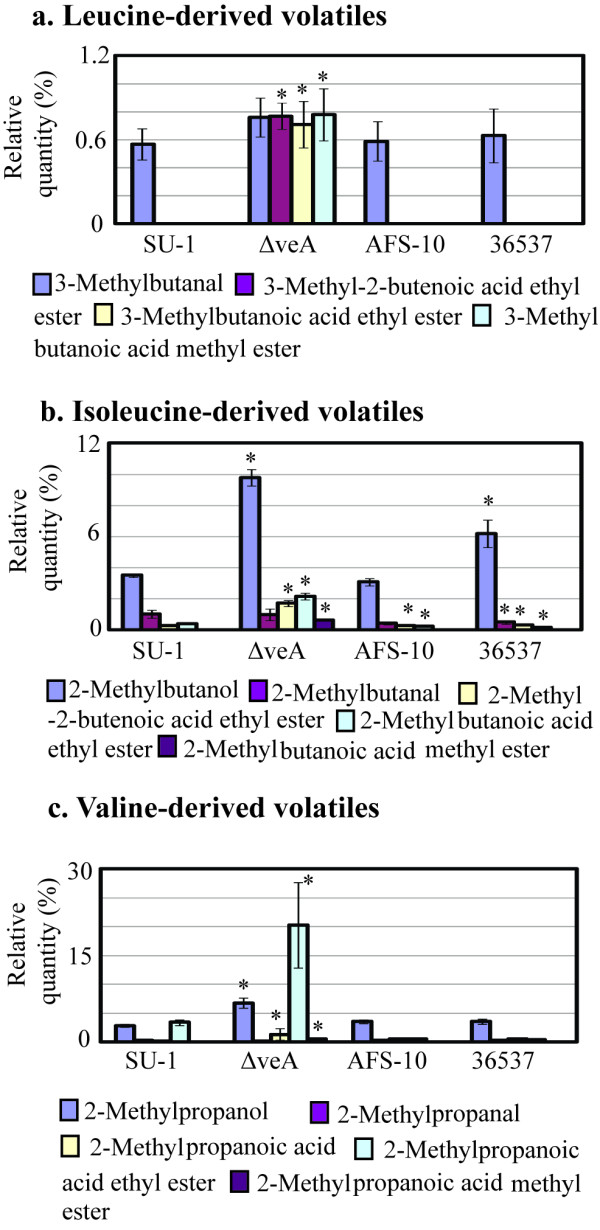
**Branched chain amino acid-derived volatiles generated by *A. parasiticus *strains grown in YES for 72 h in the light**. Conidiospores were inoculated into 100 ml of liquid YES medium at 10^4^/ml and the cultures were grown at 30°C, with shaking at 150 rpm, in the light for 72 h. Volatiles were analyzed as described in Methods. *, statistically significant difference as compared with SU-1, P < 0.01.

The volatile profile of SU-1 grown for 72 h in liquid YES (aflatoxin inducing conditions) in the dark revealed 24 putative fungal metabolites and 25 unknown compounds (Additional File 2, Figure S2). These volatiles could be divided into several classes of chemical compounds. The largest class of putative fungal metabolites included compounds derived from intermediates in metabolism of branched chain amino acids (leucine, isoleucine, and valine; see Additional File 3, Figure S3) and esters. Additional classes of compounds included alcohols (1-butanol, 1-propanol, and ethanol), lipid-derived volatiles (2-methylfuran and 1,1-diethoxy-ethane), aldehydes (formaldehyde), and organic acids (acetic acid). The relative quantities of volatiles derived from metabolism of branched chain amino acids in the dark and light were similar (Figure 1, 2). However, we observed differences in number of leucine- and valine-derived volatiles (but not isoleucine-derived volatiles) that were produced in the light versus dark. The number and relative quantities of branched chain amino acid-derived volatiles detected in the light in YES were higher at 72 h as compared with 48 h (Additional File 4, Figure S4).

We compared volatiles generated by *A. parasiticus *SU-1 grown for 72 h in the light in GMS (chemically defined medium, contains glucose) to those generated in YES. In GMS, the fungus produced a lower number of compounds of all classes of volatiles identified (including volatiles derived from branched chain amino acids and lipids) than in YES (not shown).

### A genetic block in aflatoxin biosynthesis affects the volatile profile

We compared volatiles produced by *A. parasiticus *SU-1 (wild type) and *A. parasiticus *strains impaired in aflatoxin biosynthesis, AFS10 and 36537, grown in a rich medium (YES) for 72 h in the dark. Aflatoxin synthesis is blocked in AFS10 (gene disruption in a positive pathway regulator, *aflR*; no aflatoxin enzymes or aflatoxin are synthesized) and in *A. parasiticus *ATCC36537 that carries a mutation in the aflatoxin pathway gene, *ver-*1 (accumulates the pathway intermediate versicolorin A). In the dark, AFS10 and 36537 generated similar relative quantities of 3-methylbutanal, a presumable intermediate in leucine metabolism, as compared to the wild type strain SU-1 (Figure 1a). However, no 3-methylbutanoic acid ethyl ester was produced by these two mutants (Figure 1a). None of the strains studied produced 3-methylbutanol as well.

All studied strains generated 2-methylbutanol, a putative derivative of isoleucine catabolism (Figure 1b). Nonetheless, the ethyl and methyl esters of the corresponding 2-methylbutanoic acid (2-methylbutanoic acid ethyl ester and 2-methylbutanoic acid methyl ester) were produced by the mutants in less quantity as compared with SU-1.

Accumulation of 2-methylpropanoic acid ethyl ester, a derivative of valine metabolism, was significantly reduced in AFS10 and 36537 as compared to the wild type SU-1 (Figure 1c). All strains, SU-1, AFS10, and 36537, generated 2-methylpropanol.

### Disruption of *veA *enhances accumulation of metabolites in branched chain amino acid catabolism

The volatile profile produced by Δ*veA *was significantly different than the profile of SU-1 (the wild type) and ATCC 36537 (genetic control for Δ*veA*). *A. parasiticus *Δ*veA *generated significantly higher quantities of metabolites (relative to SU-1 and 36537) derived from catabolism of the branched chain amino acids leucine, isoleucine, and valine in the dark and in the light (Figure 1, 2). For instance, quantities of the branched chain alcohols 2-methylbutanol, and 2-methylpropanol were doubled in Δ*veA*. Ethyl and methyl esters derived from branched chain amino acids (derived presumably from leucine, isoleucine, and valine) increased up to 10 fold (and higher for several compounds) as compared with SU-1 and 36537. Four esters were unique to Δ*veA *(Additional File 5, Figure S5). One of these, 2-methylbutanoic acid methyl ester is found in the aroma of gooseberry [49], which may explain the observed fruity smell of Δ*veA *cultures.

Interestingly, more than 2 fold higher quantities of ethyl acetate and acetic acid were also detected in Δ*veA *(Additional File 2, Figure S2) in comparison to 36537 and SU-1.

### Feeding *A. parasiticus *Δ*veA *with leucine, isoleucine, and valine

**Figure 3:**
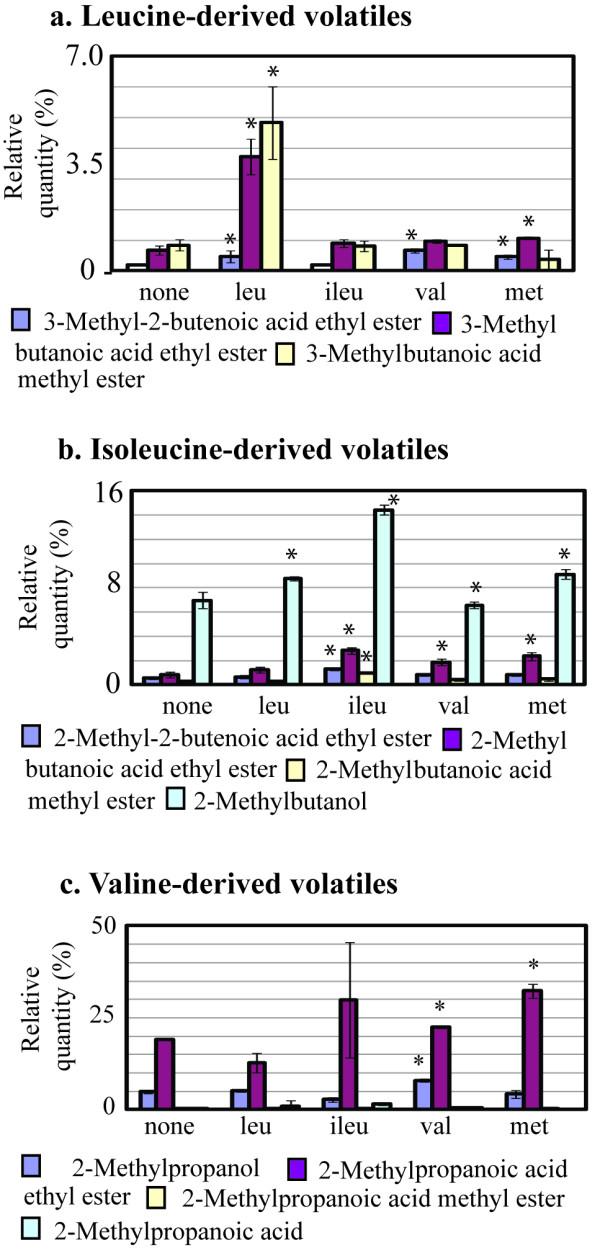
**Effect of amino acid feeding on volatile production by *A. parasiticus *Δ*veA *grown in the dark**. Conidiospores were inoculated into 100 ml of liquid YES medium at 10^4^/ml and the cultures were grown at 30°C, with shaking at 150 rpm, in the dark for 48 h. Then the amino acids were added and volatiles were analyzed after 18 h of additional incubation as described in Methods.

We determined that YES medium contains low levels of 2-methylbutanal, 3-methylbutanal, and 2-methylpropanal (not shown), which serve as precursors to synthesis of the corresponding branched chain alcohols. To examine whether the branched chain alcohols and esters generated in elevated quantities by *A. parasiticus *Δ*veA *relate to catabolism of the branched chain amino acids leucine, isoleucine and valine by the fungus, these amino acids were added to 48 h old cultures of *A. parasiticus *Δ*veA *at a final concentration of 0.03 M and the volatiles were analyzed after 18 h. Methionine (0.03 M final concentration) was added to a separate flask as a control. Feeding with leucine increased formation of the esters corresponding to leucine catabolism; however, formation of 3-methylbutanol, an expected product of leucine catabolism, was not detected either with or without addition of leucine (Figure 1a, Figure 3a). Added isoleucine and valine significantly (several fold) increased production of the expected corresponding esters and alcohols (2-methylbutanol, and 2-methylpropanol) (Figure 3b, c). Feeding with amino acids also elevated production of certain non-corresponding volatiles. For example, addition of valine increased accumulation of 3-methylbutanoic acid ethyl ester, a product of leucine catabolism. Addition of isoleucine and methionine increased formation of the products of valine catabolism including 2-methylpropanoic acid ethyl ester.

### Disruption of *veA *increases ethanol production by *A. parasiticus ΔveA*

It was shown previously that aspergilli can produce ethanol [50]. In that study, an inverse regulatory relationship between aflatoxin and ethanol accumulation was demonstrated. Aflastatin A, an inhibitor of aflatoxin production, was shown to inhibit aflatoxin biosynthesis and concurrently to inhibit ethanol catabolism at the transcriptional level thus resulting in an increase of ethanol accumulation by *A. parasiticus*; glucose consumption also increased [50,51].

**Figure 4:**
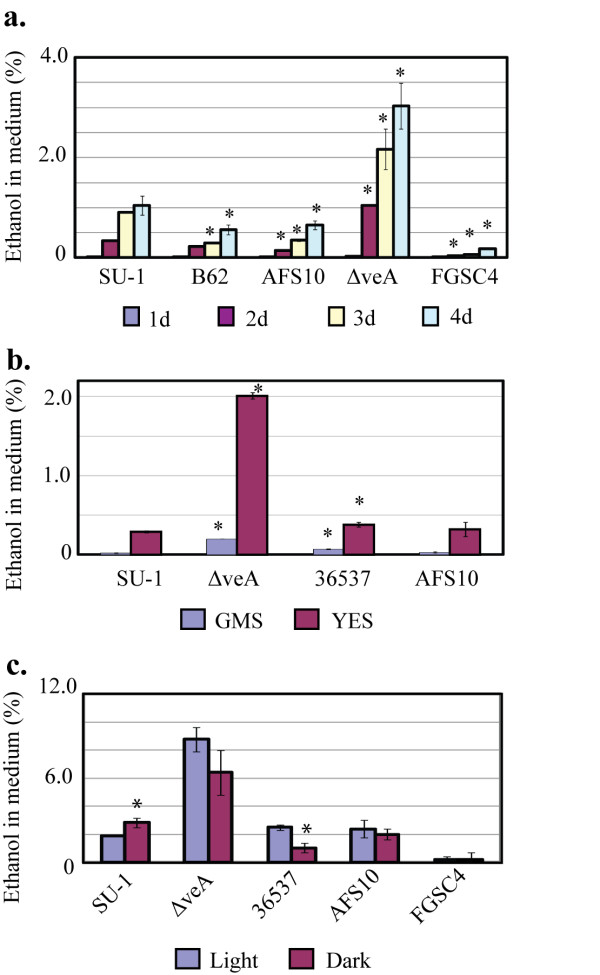
**Ethanol production by aspergilla**. Ethanol levels were measured using GC as described in Methods. **A**. Aspergilli were grown in YES liquid medium in the dark for 4 days. **B**. *A. parasiticus *strains were grown in YES or GMS liquid media in the light for 72 h. **C**. Aspergilli were grown inYES liquid media in the light and in the dark for 72 h. *, statistically significant difference as compared with SU-1, P < 0.01.

We demonstrated that in YES, *A. parasiticus *strains including SU-1, B62 (*nor-1 *mutant, accumulates the pathway intermediate norsolorinic acid), AFS10, and Δ*veA *produced significantly higher quantities of ethanol than *A. nidulans *FGSC4 at each time point tested (the experiment was performed in the dark for 4 days) (Figure 4a). In the chemically defined medium GMS, all *A. parasiticus *strains tested (SU-1, AFS10, 36537, and Δ*veA*) generated several fold lower quantities of ethanol as compared with YES medium (Figure 4b). Light did not influence ethanol production by either strain of *A. parasiticus *(Figure 4c). Feeding with leucine (as described above) did not significantly affect production of ethanol by the wild type SU-1 (not shown).

A genetic block in aflatoxin biosynthesis in AFS10 or in 36537 resulted in a decreased formation of ethanol by these mutant strains in comparison to SU-1. However, disruption of *veA *resulted in 3 to 4 fold higher levels of ethanol as compared to SU-1, or 36537 (Figure 4a, b, c) under all conditions tested; the concentration of ethanol in the Δ*veA *culture medium ranged from 2 to 8%. Feeding with leucine and valine (as described above) did not significantly affect production of ethanol by Δ*veA *(not shown). However, isoleucine feeding resulted in a slight inhibition of ethanol production (not shown).

### Volatiles produced by *A. parasiticus *Δ*veA *affect conidiospore and sclerotia formation

**Figure 5:**
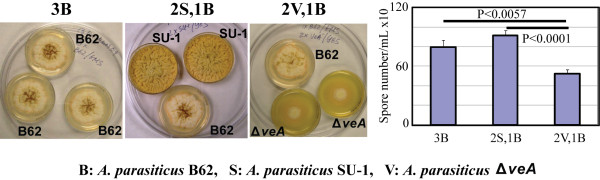
**Effect of fungal volatiles on *A. parasiticus *B62 sporulation**. Spores were center inoculated on agar media and grown for 5 days at 30°C in the dark. B62 was grown on GMS while SU-1 and Δ*veA *were grown on YES agar medium. Small Petri dishes containing colonies (no lids) were arranged within large Petri dishes as shown above. B62 conidia are represented by dark brown dots in the center of colonies.

Disruption of *veA *results in developmental defects (blocks asexual conidiation in the dark and sclerotia formation [32,52]). We previously showed that fungal volatiles play a role in the control of secondary metabolism [53]. To test whether the volatiles produced by Δ*veA *participate in the molecular machinery that regulates aflatoxin biosynthesis and asexual conidiation, we grew *A. parasiticus *B62 (accumulates the red colored aflatoxin intermediate norsolorinic acid along the colony margin) on agar medium in the presence of volatiles generated by Δ*veA*. We observed an approximately 35% to 55% reduction in conidiation in B62 after exposure to Δ*veA *volatiles for 5 days (Figure 5). Accumulation of norsolorinic acid was not affected (not shown).

To analyze the effect of Δ*veA *volatiles on sclerotia formation, *A. parasiticus *SU-1 and ATCC 36537 were grown on coconut or YGT agar media (both media were previously shown to induce sclerotia formation [22,32]) in the dark in the presence of Δ*veA *volatiles (see Methods). *A. parasiticus *SU-1 grown on coconut agar medium for 9 days demonstrated an approximately 30 to 40% decrease in the number of sclerotia in the presence of Δ*veA *volatiles (Table 2). However, no significant effect on the number of sclerotia formed on YGT was observed (not shown). Under all conditions tested, sclerotia were black in color and were able to produce colonies after harvest followed by inoculation onto YES agar medium. Interestingly, SU-1 conidiospores that developed on coconut medium in the presence of Δ*veA *volatiles for 17 days were dark brown, whereas conidiospores developed under SU-1 volatiles were dark green indicating that volatiles also may affect biosynthesis of conidial pigment.

**Table 2:** Volatiles generated by *A. parasiticus *Δ*veA *reduce sclerotia production by SU-1 grown on coconut agar medium

Sclerotia developed by SU-1 on coconut agar medium; lid #	Volatiles were produced by aspergilli grown on YES or coconut agar medium, or YES agar medium only (two lids of each)			
	
	Δ*veA *on YES	SU-1 on coconut	SU-1 on YES	YES only
1	231	397	252	493

2	231	364	314	258

3	232	618	n\e	307

Three 60 × 15 mm Petri dish lids were placed inside a large 150 × 15 mm Petri dish as described in Methods. For sclerotia development 10^4^spores of *A. parasiticus *SU-1 were center inoculated onto one lid that contained coconut agar medium. For volatiles generation, two other lids contained either Δ*veA *inoculated on YES agar medium, or SU-1 inoculated on coconut agar medium (control), or SU-1 inoculated on YES agar medium (control), or YES agar medium only (control). The cultures were grown in the dark at 30°C for 9 days. The experiment was performed in triplicate. The number of sclerotia developed by SU-1 grown on coconut agar medium per plate is presented. n\e, not estimated.

### Analysis of transcript accumulation for branched chain amino acid aminotransferase and alcohol dehydrogenase

**Figure 6:**
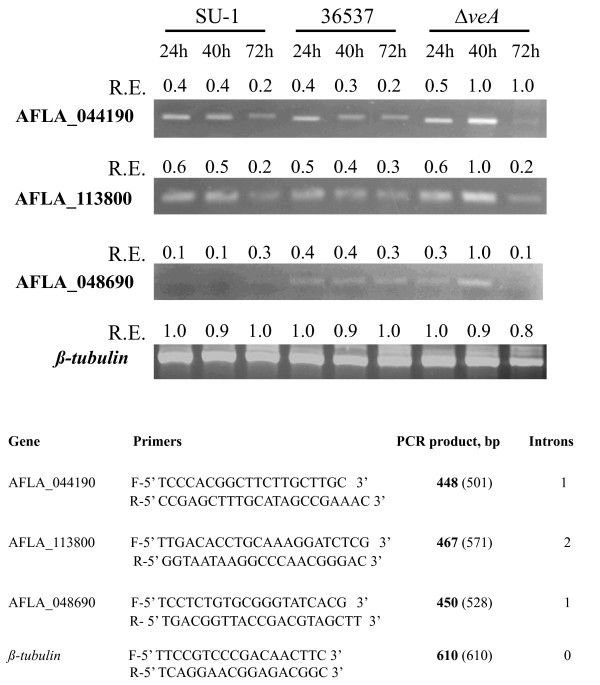
**Relative expression of AFLA_044190, AFLA_113800 and AFLA_048690 in SU-1, ATCC36537 and *ΔveA***. For growth conditions and RT-PCR methods, see Methods. Intensities of the PCR bands obtained for each time point (24 h, 40 h and 72 h) for a particular gene were compared. Relative intensity (R.I) for a band (or relative expression, R.E.) is reported as the ratio of the absolute intensity of the band to the highest absolute intensity recorded for any time-point. Absolute intensity values were measured with Adobe Photoshop software. The number in parenthesis indicates the expected size of PCR product obtained using genomic DNA.

The first reaction in the catabolism of branched chain amino acids is catalyzed by a branched chain amino acid aminotransferase that forms a 2-ketoacid; this reaction controls the flow of carbon through the catabolic pathway [54]. The resulting 2-ketoacid can then be transformed into a branched chain alcohol (after decarboxylation in the presence of 2-keto acid decarboxylase), and/or into ethyl or methyl esters (see schematic in Additional File 3, Figure S3). In order to examine possible mechanisms that generate the observed elevation in the accumulation of catabolic products of branched chain amino acids, the expression of branched chain amino acid aminotransferase gene expression was analyzed. The genome of *A. flavus*, a close relative of *A. parasiticus *[12,14], contains two genes (AFLA_113800 and AFLA_044190) that encode proteins that exhibit a high percentage identity with the *Saccharomyces cerevesiae *branched chain amino acid aminotransferases BAT1 (mitochondrial) and BAT2 (cytosolic). AFLA_113800 exhibits 61% identity to *S. cerevisiae *branched chain amino acid aminotransferase BAT1 and 60% identity to BAT2 (Additional File 6, Figure S6). AFLA_044190 is 43% identical to BAT1 and 44% identical to BAT2. The expression of AFLA_113800 and AFLA_044190 was detected in SU-1 and 36537 (Figure 6). Interestingly, in Δ*veA *the expression levels for these genes were approximately 2 fold higher at 40 h as compared with SU-1 and 36537 (Figure 6); at this time point, aflatoxin biosynthesis peaks in SU-1. However, there were no significant differences in the relative concentrations of branched amino acids in SU-1, Δ*veA*, 36537, and AFS10 cultures grown for 72 h in YES (not shown).

Since we observed a significant increase in the level of ethanol accumulation in Δ*ve*A, we analyzed the expression of a gene encoding alcohol dehydrogenase, AFLA_048690. This gene exhibits the highest sequence identity (57%) with *S. cerevesiae adh1*, a gene that encodes an alcohol dehydrogenase (Additional File 7, Figure S7). *adh1 *accounts for the majority of alcohol dehydrogenase activity in baker's yeast and primarily is responsible for ethanol formation [55]. AFLA_048690 also exhibits 50 to 55% identity to the yeast genes *adh *2, 3, and 5. The yeast genes *adh*1, 2, 3, and 5 are also known to participate in the catabolism of amino acids to produce branched chain alcohols [56]. In Δ*veA *the expression level for AFLA_048690 was significantly higher at 40 h as compared with SU-1 and 36537 (Figure 6); the same pattern of expression was observed for the putative branched chain amino acid transferases AFLA_044190 and AFLA_113800 (see above, Figure 6). These results strongly suggest that VeA negatively regulates the formation of branched chain amino acid-derived volatiles and ethanol as the cells trigger secondary metabolism.

### VeA is a positive regulator of mitochondrial and peroxisomal β-oxidation

β-oxidation of fatty acids is one source that supplies precursors for polyketide biosynthesis; in addition, β-oxidation of odd number fatty acids generates propionyl-CoA that can affect the activity of a polyketide synthase involved in sterigmatocystin biosynthesis [57,58], thus presumably contributing to the Δ*veA *phenotype. Propionate is also a product of catabolism of several amino acids, including valine and isoleucine. The inability of null mutants Δ*veA *and Δ*laeA *to grow on peanut and maize seeds [59] may be explained by the failure of the mutants to metabolize host lipids due to defects in β-oxidation.

**Figure 7:**
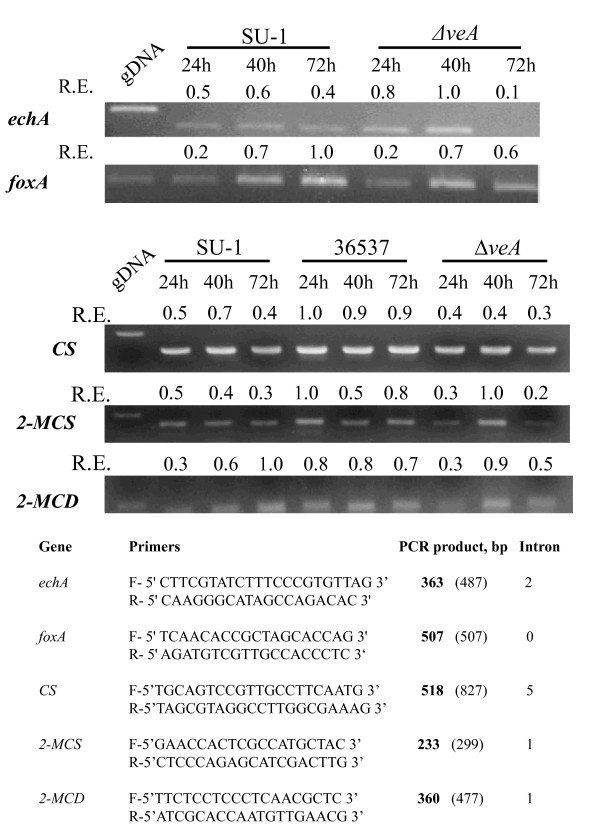
**Transcript analysis of genes involved in the methylcitrate cycle and β-oxidation of fatty acids in *A. parasiticus *strains**. For growth conditions and RT-PCR methods, see Methods. R.E., relative expression was calculated as shown in Figure 6. *CS*, citrate synthase AFLA_007020; 2-*MCS*, 2-methylcitrate synthase AFLA_049290; 2-*MCD*, 2-methylcitrate dehydratase AFLA_056350; *echA*, a short chain enoyl-CoA hydratase AFLA_043610; *foxA*, an enoyl-CoA hydratase/hydroacyl-CoA dehydrogenase AFLA_041590. The number in parenthesis indicates the expected size of PCR product obtained using genomic DNA.

We focused our attention on the genes *echA *and *foxA*, which encode, respectively, a short chain enoyl-CoA hydratase (EchA) involved in β-oxidation in mitochondria, and a multifunctional enzyme FoxA (possesses enoyl-CoA hydratase and hydroacyl-CoA dehydrogenase activities) involved in β-oxidation of long chain fatty acids in peroxisomes; these genes previously were shown to be involved in β-oxidation in *A. nidulans *[25,38]. A BLAST search using sequences of *A. nidulans foxA *and *echA *identified two homologous genes in the genome of *A. flavus*, a close relative of *A. parasiticus *[12,14]. AFLA_041590 has 81% identity to *A. nidulans foxA*; AFLA_043610 has 83% identity to *A. nidulans echA*. To analyze transcript accumulation in *A. parasiticus*, primers were designed based on the *A. flavus *gene sequences (Figure 7).

Expression of both genes in the wild type SU-1and in Δ*veA *increased from 24 h to 40 h of growth (Figure 7). By 72 h of growth we observed a decline in *echA *transcript accumulation in SU-1 and Δ*veA*; however the decrease in Δ*veA *was more severe than in SU-1. By 72 h of growth, transcript accumulation of *foxA *in SU-1 continued to increase, whereas in Δ*veA *accumulation of the *foxA *transcript declined slightly.

The methylcitrate cycle is one biochemical pathway for propionate metabolism in fungi [60-63]. We hypothesized that impairment of methylcitrate cycle would increase formation of corresponding valine- and leucine-derived esters. We examined transcript accumulation of the first two genes of the methylcitrate cycle, 2-methylcitrate synthase (2-*MCS*) and 2-methylcitrate dehydratase (2-*MCD*), in *A. parasiticus *strains (Figure 7); we also compared their pattern of accumulation with transcript accumulation of citrate synthase. We detected transcripts for all three genes in all strains tested. However, in Δ*veA*, transcripts for 2-MCS and 2-MCD increased at 30 h and declined by 40 h, in contrast to the wild type SU-1, which showed a slight decrease in transcript accumulation for 2-MCS from 24 h to 40 h. These data suggest that the 2-methylcitrate cycle is not impaired in Δ*veA*.

## Discussion

A metabolomics approach previously was used to link complex biochemical and cellular functions to genomics in plants and yeast [45,64-66]. However, relatively few (and incomplete) metabolomics studies have been reported for filamentous fungi [3,67-70]. Volatile compounds represent a significant portion of the metabolome and, as has been demonstrated in our study, they provide information on the real-time metabolic changes that occur within the fungal cell; most importantly from a practical stand point, this approach does not require quantification of the metabolites or cell disruption. We show that SPME-GC/MS is a sensitive, fast, and accurate approach to study changes in volatile compounds generated by the filamentous fungi.

Our studies demonstrate that *A. parasiticus *produces a variety of volatile organic compounds including several classes of intermediates and products associated with catabolism of the branched chain amino acids (leucine, isoleucine, and valine) and lipids; alcohols, organic acids, esters, and aldehydes were also detected. Our data suggest that *A. parasiticus *catabolizes free branched chain amino acids (endogenously synthesized, or exogenously added); these may either enter the catabolic pathway directly, or they could be used as a carbon source, thus producing detectable levels of metabolic cross talk.

2-ketoacids are also synthesized *de novo *as late intermediates in branched chain amino acid biosynthesis pathways; they can be converted to the corresponding alcohols and esters. Our feeding studies provide evidence that fungal volatile compounds originate from branched chain amino acids catabolism; however, we can not rule out the possibility that 2-ketoacids generated *de novo *through biosynthetic pathway serve as an additional precursor for volatile synthesis.

We also demonstrate that the number of volatile compounds produced in culture depends on the composition of the growth medium, the presence or absence of light, and also on the status of secondary metabolism in the fungal cell. A genetic block in aflatoxin biosynthesis in AFS10 and ATCC 36537 resulted in a decrease in formation of isoleucine- and valine-derived acids and esters; intermediates in leucine catabolism (except for 3-methylbutanal) were barely detected. These data support the idea that secondary metabolism (aflatoxin biosynthesis) is an integrated part of the cellular metabolism.

Our data show that disruption of secondary metabolic pathways in Δ*veA *correlate with dramatic changes in carbon flow through primary metabolic pathways. The most significant metabolic changes were observed in catabolism of branched chain amino acids and formation of ethanol in *A. parasiticus*; the data strongly suggest that VeA acts as a negative regulator of these processes at the transcriptional level. Based on previous and current work, we propose a model for the association between secondary metabolism and catabolism of branched chain amino acids and ethanol biosynthesis in *A. parasiticus*; the model proposes that Velvet A plays a key regulatory role in the coordination of carbon flow through these metabolic processes (Figure 7).

How can one explain the changes in carbon flow observed in Δ*ve*A? Under conditions studied (liquid shake culture, no development occurs) disruption of *veA *is thought to impair most if not all of secondary metabolism [32,52]. Thus it is reasonable to suggest that the cell must re-structure and re-direct its metabolism and carbon flow in order to maintain cellular homeostasis. An increase in production of ethanol and branched chain alcohols and esters may serve as a compensatory mechanism to maintain cellular redox balance and to promote carbon removal from the cell. Our data suggest that, at least at the transcription level, accumulation of acetyl-CoA and propionyl-CoA in SU-1 are balanced by channeling through polyketide biosynthesis, the methylcitrate cycle, and ethanol formation. At 40 h, the Δ*veA *strain, which is aberrant in secondary metabolism, compensates for the increase in accumulation of propionyl-CoA by increasing methylcitrate cycle activity as well as the formation of ethyl-propionate and corresponding esters. Acetyl-CoA is re-directed through ethanol biosynthesis. The block in secondary metabolism in Δ*veA*, also directs carbon flow through formation of branched chain acyl-CoA-derived alcohols, acids and esters. At 72 h, these compensatory mechanisms in Δ*veA *likely discontinue resulting in the accumulation of acetyl-CoA and propionyl-CoA, and in the activation of a feed-back inhibition mechanism that affects β-oxidation both in mitochondria and peroxisomes. In this scenario, VeA controls β-oxidation indirectly through accumulation of acetyl-CoA and propionyl-CoA. However, whether the effect of VeA on gene transcription is mediated directly through protein-protein interactions, indirectly through intracellular biochemical changes, or both, remains to be elucidated.

**Figure 8:**
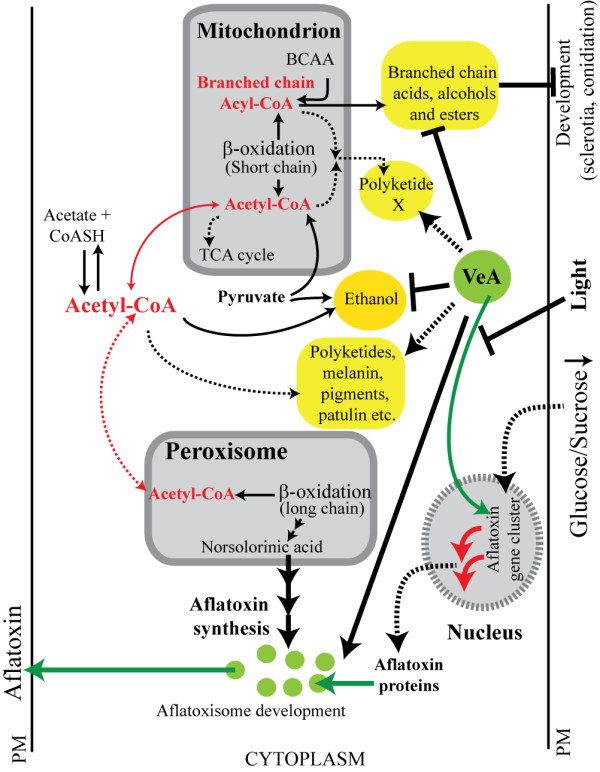
**VeA controls intracellular carbon flow in *Aspergillus parasiticus***. The schematic illustrates compartmentalization of biosynthesis of secondary metabolites, branched chain amino acid catabolism, and biosynthesis of ethanol in *Aspergillus*. Acetyl-CoA is produced in the mitochondrion, the peroxisome and in the cytoplasm; acetyl-CoA is the precursor of aflatoxin and other secondary metabolites [4]. Early steps in aflatoxin biosynthesis occur in peroxisomes [22]; the middle and late steps take place in aflatoxisomes [4,12]. The biosynthesis and catabolism of branched chain amino acids occur in the mitochondrion; branched chain acyl-CoAs serve as the precursors of branched chain acids, branched chain alcohols and branched chain esters. Acyl-CoA and acetyl-CoA serve as the precursors of the unknown polyketide X. Ethanol is produced through nonoxidative decarboxylation of pyruvate followed by conversion of acetaldehyde to ethanol by alcoholdehydrogenase; acety-CoA may also be converted to ethanol. VeA negatively regulates branched chain amino acid catabolism and ethanol biosynthesis. In addition, VeA is a positive regulator of β-oxidation of fatty acids in mitochondria and peroxisomes during the late stages of stationary phase. When secondary metabolism is blocked in Δ*veA*, carbon flow is re-directed to elevated ethanol production and branched chain amino acid-derived volatiles. Overall, VeA is "a master-coordinator", which plays a role in regulation of carbon flow through metabolic processes (primary and secondary) in different cellular compartments. Known metabolic and regulatory pathways are shown by solid lines; hypothesized pathways are indicated by dashed lines. Abbreviations: BCAA, branched chain amino acids; PM, plasma membrane.

One alternative explanation for the observed changes in carbon flow in Δ*ve*A relates to studies on the biosynthesis of polyketide antibiotics by *Streptomyces spp*. The polyketides virginiamycin, tautomycin, mananumycin, butyrolactols, and antraquinones are generated by condensation of starter units including isobutyryl-CoA, isovaleryl-CoA, and 2-methylbutyryl-CoA; the latter are derived from catabolism of the branched chain amino acids valine, leucine, and isoleucine respectively [71,72]. We propose that *Aspergillus spp*. synthesize polyketide(s) using branched acyl-CoA as precursors that originate through catabolism (or biosynthesis) of branched chain amino acids and or β-oxidation of fatty acids, and that *veA *positively regulates this biosynthetic pathway. Maggio-Hall et al. [39] provided evidence that mitochondrial β-oxidation of fatty acids and catabolism of branched chain amino acids utilize the same acyl-CoA dehydrogenase encoded by *scdA*. We hypothesize that, in the presence of VeA, β-oxidation of fatty acids and catabolism of branched chain amino acids occur in mitochondria and provide the necessary starter units for biosynthesis of polyketides, similar to the situation observed in *Streptomyces spp*. In Δ*ve*A, the biosynthesis of these polyketide(s) is blocked, which causes a shift in metabolism to stimulate accumulation of branched chain alcohols and branched chain esters (Figure 8). The regulatory feed back mechanisms underlie the decline in β-oxidation of fatty acids. Interestingly, valine is synthesized also in the mitochondrion [73]. Alternatively, *A. parasiticus *produces other secondary metabolites derived from branched chain amino acids. Future work is focused on testing the hypothesis stated above.

VeA is a global regulator of morphogenesis and secondary metabolism in *Aspergillus spp *[12,24,32,52]; this gene is involved in transcriptional regulation of several hundred genes [74]. Our work provides additional mechanistic details about the molecular machinery by which VeA regulates gene expression and therefore conidiation and sclerotia formation. We demonstrated that deletion of *veA *results in accumulation of volatile compounds with biological activity, which in turn, participate in the regulation of developmental processes. What is the role of LaeA that forms a protein complex with VeA in this regulation? Future studies are necessary to better understand the mechanisms that underlie this phenomenon.

## Conclusions

1) SPME-GC/MS volatile profiling analysis is a powerful approach to identify intracellular metabolic changes and the direction of carbon flow in filamentous fungi. An important practical advantage of this approach is that there is no need to calculate individual metabolite concentration or to disrupt the cells. 2) VeA coordinates biosynthesis of secondary metabolites with catabolism of branched chain amino acids and alcohol biosynthesis. 3) Our work provides insight on how changes in intracellular chemical development are linked to morphological development. 4) Understanding carbon flow through secondary metabolic pathways and catabolism of branched chain amino acids is essential for controlling and customizing production of natural products.

## Methods

### Strains, growth media, and growth conditions

The isogenic *A. parasiticus *strains used in this study were derived from SU-1 (ATCC 56775), a wild type aflatoxin producer (Table 1). AFS10 is an aflatoxin non-producing strain derived from the parent strain, SU-1; gene disruption of *aflR *in AFS10 blocks aflatoxin synthesis and expression of several aflatoxin genes. AFS10 was kindly provided by Dr. J. Cary [33,75]. *A. parasiticus *ATCC36537 (*ver-1*, *wh-1*) was generated from *A. parasiticus *SU-1 by U.V. irradiation [76]. This strain accumulates the aflatoxin pathway intermediate versicolorin A due to a point mutation in Ver-1A at nucleotide residue 287 (G to A) thus resulting in a non-functional enzyme [77]. The *veA *deletion strain *A. parasiticus *Δ*veA *(*ver-1, wh-1, pyrG, ΔveA::pyrG*) was generated from *A. parasiticus *CS10 (*ver-1, pyrG, wh-1*) by a double-crossover event exchanging the *pyrG *selectable marker for the *veA *coding region [32]. CS10 was in turn generated from *A. parasiticus *ATCC36537 by spontaneous mutation using N-methyl-N'-nitro-N-nitrosoguanidine followed by enzymatic analysis [78].

YES liquid medium (contains 2% yeast extract and 6% sucrose; pH 5.8) was used as an aflatoxin inducing growth medium. A chemically defined glucose minimal salts (GMS) medium supplemented with 5 μM Zn^2+ ^was prepared as described elsewhere [79]. YGT medium (0.5% [wt/vol] yeast extract, 2% [wt/vol] glucose, and 1 ml of trace element solution per liter of medium) was prepared as described previously [32]. Coconut agar medium was prepared as described by Mahanti et al. [22]. 10^4 ^spores/ml were inoculated into liquid medium.

To analyze the effect of fungal volatiles on aflatoxin biosynthesis and fungal development, the fungus was grown in 60 × 15 mm Petri dish lids. 10^4 ^spores were center inoculated onto the agar medium. Three lids were placed inside a larger, 150 × 15 mm Petri dish as described previously [53]. This system allowed free gas and volatile exchange between colonies inside the large dish while preventing direct colony contact.

Growth of *A. parasiticus *strains was estimated by dry weight of the mycelia. Mycelia were harvested at appropriate times of growth by filtration through Miracloth (Calbiochem/EMD Biosciences, La Jolla, CA) and dried for 48 h at 90°C.

### Detection of aflatoxin B_1_, B_2_, G_1_, G_2 _and norsolorinic acid

Aflatoxins in the agar medium and mycelium were extracted 3 times with 5 ml chloroform (15 ml total). The extracts were dried under a stream of N_2 _and re-dissolved in 70% methanol. Aflatoxins were detected by TLC and ELISA as described by Roze at al. [80]. ELISA provided an estimation of AFB_1 _levels, whereas TLC enabled one to estimate levels of AFB_1_, AFB_2_, AFG_1_, and AFG_2_. Norsolorinic acid was extracted from the agar and mycelium with chloroform and then acetone, and its quantity was analyzed by TLC [81].

### Evaluation of conidiation

*A. parasiticus *conidia were harvested and their number per colony was estimated as described by Roze et al. [80].

### Volatile compound analysis by SPME-GC/MS

Sampling and volatile analysis were performed essentially as described previously [42] with minor modifications. *Sample preparation and SPME analysis*. Twelve ml of fungal culture were harvested at regular intervals and dispensed into 22 ml clear screw cap vials equipped with Mininert^® ^Valves (all from Supelco, Bellefonte, PA). Vials with cultures were pre-equilibrated at 30°C (the same temperature we used for fungal growth) in a water bath for at least 30 min, shaking at 50 rpm, before headspace gases were sampled at 30°C. A 65 μm PDMS/DVB SPME fiber (Supelco) was conditioned at 250°C overnight. Sampling was performed at 30°C by placing the fiber through the Mininert Valve into the headspace above the fungal culture for 3 min. Vials were continuously swirled at 100 rpm during incubation and SPME exposure. *GC/MS parameters*. Volatiles were desorbed from the fiber in a gas chromatograph (HP-6890, Hewlett-Packard Co., Wilmington, DE) injection port for 3 min; absorption and desorption time was optimized as described in [42]. Volatiles were separated on a 29 m/250 μm i.d. capillary column HP-5MS (Hewlett-Packard Co., Wilmington, DE) having a film thickness 0.25 μm. The first 20 cm of the column was cooled with liquid nitrogen during the desorption process to cryofocus the volatiles. Ultrapure helium (99.999%) was used as a carrier gas at a flow rate of 1.5 ml/min. The initial temperature of the column (40°C) was increased upon removal of liquid nitrogen at 60°C/min to obtain a final temperature of 250°C, which was maintained for 1 min. Following chromatographic separation, metabolites were fragmented with an electron ionization source and ion masses were detected by time-of-flight mass spectrometry (FCD-650, LECO Corp., St. Joseph, MI). Preliminary identification of metabolites was achieved by comparison of their mass spectra with those of authenticated chemical standards contained in a mass spectrum library (National Institute for Standard Technology, Search Version 1.5, Gaithersburg, MD). A total of 4 to10 biological replicates were performed for each strain and condition. Only compounds detected in 50% or more replicates were then confirmed by comparison of their GC retention time, MS ion spectra and retention index (RI). Finally, the compounds with probability values below 70% were rejected.

### Ethanol measurements

Twelve ml of fungal culture were dispensed into 22 ml clear screw cap vials equipped with Mininert^® ^Valves (all from Supelco). Vials were incubated at 30°C for at least 30 min before headspace gases were sampled and ethanol levels were determined by means of gas chromatography (GC) using ethanol standards as described previously [80].

### Assessment of sclerotia production

Small (60 × 15 mm) agar plates were center-inoculated with 10^4 ^conidiospores and placed into a large Petri dish (150 × 15 mm) as described above. The cultures were incubated at 30°C in the dark at 90% relative humidity. After 9 to 17 days, the colonies were sprayed with 95% ethanol to enhance visualization of sclerotia. The number of sclerotia per plate was assessed. The viability of sclerotia was tested by placing 5 randomly chosen sclerotia onto YES agar medium which was incubated for 7 days in the dark.

### Feeding of branched chain amino acids

Conidiospores (10^4^/ml) were inoculated into YES liquid medium and incubated for 48 h at 30°C as described above. Sterile solutions of L-leucine, L-isoleucine, or L-valine (all from Sigma, St. Louis, MO) in YES liquid medium were added to a final concentration of 0.03 M and incubation continued for an additional 18 h. Analysis of volatiles was conducted as described above.

### Analysis of leucine, isoleucine, and valine accumulation using LC/MS/MS

Cultures were inoculated into YES liquid medium and incubated for designated periods of time at 30°C as described above. 1 ml of each culture (containing medium plus mycelia) was extracted with 10 ml solvent (acetonitril:isopropanol:water = 3:3:2) for 1 h at RT. The extract was filtered through Whatman #1 filter paper, then through a 0.45 μm sterile filter (MILLEX^® ^HA, Millipore, Carrigtwohill, Co. Cork, Ireland); the extract was stored at -20°C. Ten μl of each extract were analyzed by a 3200 Q-Trap LC/MS/MS system (Applied Biosystems, Foster City, CA) at the RTSF/Mass Spectrometry Facility, MSU, using a ZIC-pHILIC column (SeQuant Merck, Darmstadt, Germany); acetonitril and 10 mM ammonium acetate in H_2_O were used as solvents with gradients of acetonitrile 98%, 50%, 5%.

### Analysis of gene expression using RT-PCR

Total RNA extraction and preparation of cDNA was conducted as described elsewhere [14]. Primers (Figure 6) were designed based on an *A. flavus *genome database http://www.aspergillusflavus.org; the *A. flavus *genome exhibits 95-98% similarity to the *A. parasiticus *genome [12,14].

## Abbreviations

SPME-GC/MS: solid phase microextraction - gas chromatography/mass spectrometry; NIST: National Institute for Standard Technology; YES: yeast extract sucrose; GMS: glucose minimal salts.

## Authors' contributions

LVR and JEL designed research; LVR, AC, AAK, AVK, DA, and DV performed research; ML, RMB, and ADJ contributed new technology/analytic tools; LVR and AVK analyzed the data; LVR and JEL wrote the paper. All authors read and approved the final manuscript.

## Supplementary Material

Additional file 1 **Figure S1 - Growth of *A. parasiticus *strains in YES liquid medium**. Conidiospores were inoculated into 100 ml of liquid YES medium at 10^4^/ml and the cultures were grown at 30°C, with shaking at150 rpm, in the dark for designated periods of time. Dry weight was estimated as described in Methods. Click here for file

Additional file 2 **Figure S2 - SPME-GC/MS headspace gas analysis of selected volatile compounds produced by aspergilli grown in YES medium in the dark for 72 h**. Conidiospores were inoculated into 100 ml of liquid YES medium at 10^4^/ml and the cultures were grown at 30°C, with shaking at 150 rpm, in the dark for 72 h. Each culture was grown in two individual flasks. Each experiment was conducted in triplicate. The results are presented as an average of six measurements of relative peak area × 10^4 ^+ S.E. R.T., retention time, sec. Click here for file

Additional file 3 **Figure S3 - Production of fungal volatiles through pathways of branched chain amino acid catabolism**. 2-ketoacids, the main intermediates, are formed through enzymatic transamination of branched chain amino acids; they can also be synthesized *de novo*. 2-keto acid decarboxylase leads to formation of the corresponding alcohols. 2-ketoacid dehydrogenase leads to formation of the corresponding CoA derivatives and, subsequently to methyl and ethyl esters. Click here for file

Additional file 4 **Figure S4 - Branched chain amino acid-derived volatiles generated by SU-1 grown for 48 h and 72 h in light**. Conidiospores were inoculated into 100 ml of liquid YES medium at 10^4^/ml and the cultures were grown at 30°C, with shaking at150 rpm, in the light for 48 h and 72 h. Volatiles were analyzed as described in Methods. Click here for file

Additional file 5 **Figure S5 - Branched chain amino acid-derived esters detected in *A. parasiticus *strains**. *, esters unique to Δ*veA*. Click here for file

Additional file 6 **Figure S6 - Amino acid sequence alignment of the putative *A. flavus *branched chain amino acid aminotransferases AFLA_113800 and AFLA_044190 with yeast BAT1 and BAT2**. Amino acid sequences were aligned using Clustal multiple sequence alignment program. AFLA_113800 exhibits 61% identity to BAT1 and 60% identity to BAT2. AFLA_044190 exhibits 43% identity to BAT1 and 44% identity to BAT2. The highlighted lysine residue represents the active site of the protein in *E.coli*. An *asterisk *was added below the sequences at conserved amino acid. Click here for file

Additional file 7 **Figure S7 - Amino acid sequence alignment of the putative *A. flavus *alcohol dehydrogenase, AFLA_048690, with the yeast alcohol dehydrogenase, ADH1**. Amino acid sequences were aligned using Clustal multiple sequence alignment program. AFLA_048690 exhibits 57% identity to yeast ADH1. An *asterisk *was added below the sequences at conserved amino acid. Click here for file
