# Supplementary figures and images for: Volatile profiling reveals intracellular metabolic changes in Aspergillus parasiticus: veA regulates branched chain amino acid and ethanol metabolism
Source: BMC Biochem. 2010 Aug 24;11:33. doi: 10.1186/1471-2091-11-33 (PMC2939540; doi:10.1186/1471-2091-11-33)

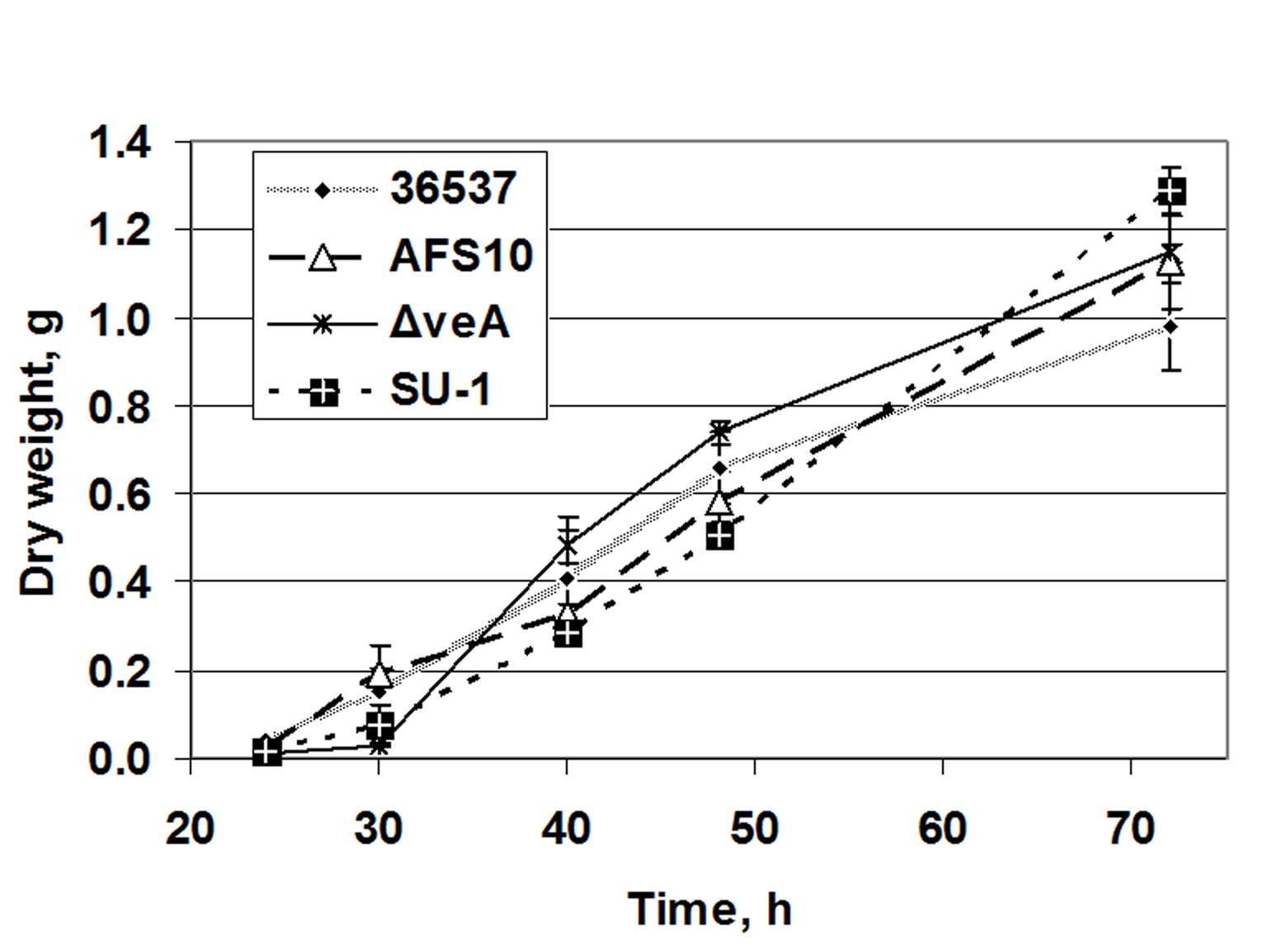

Supplement: Additional file 1 — Figure S1 - Growth of A. parasiticus strains in YES liquid medium. Conidiospores were inoculated into 100 ml of liquid YES medium at 104/ml and the cultures were grown at 30°C, with shaking at150 rpm, in the dark for designated periods of time. Dry weight was estimated as described in Methods. [file 1471-2091-11-33-S1.TIFF]

## Slide 1
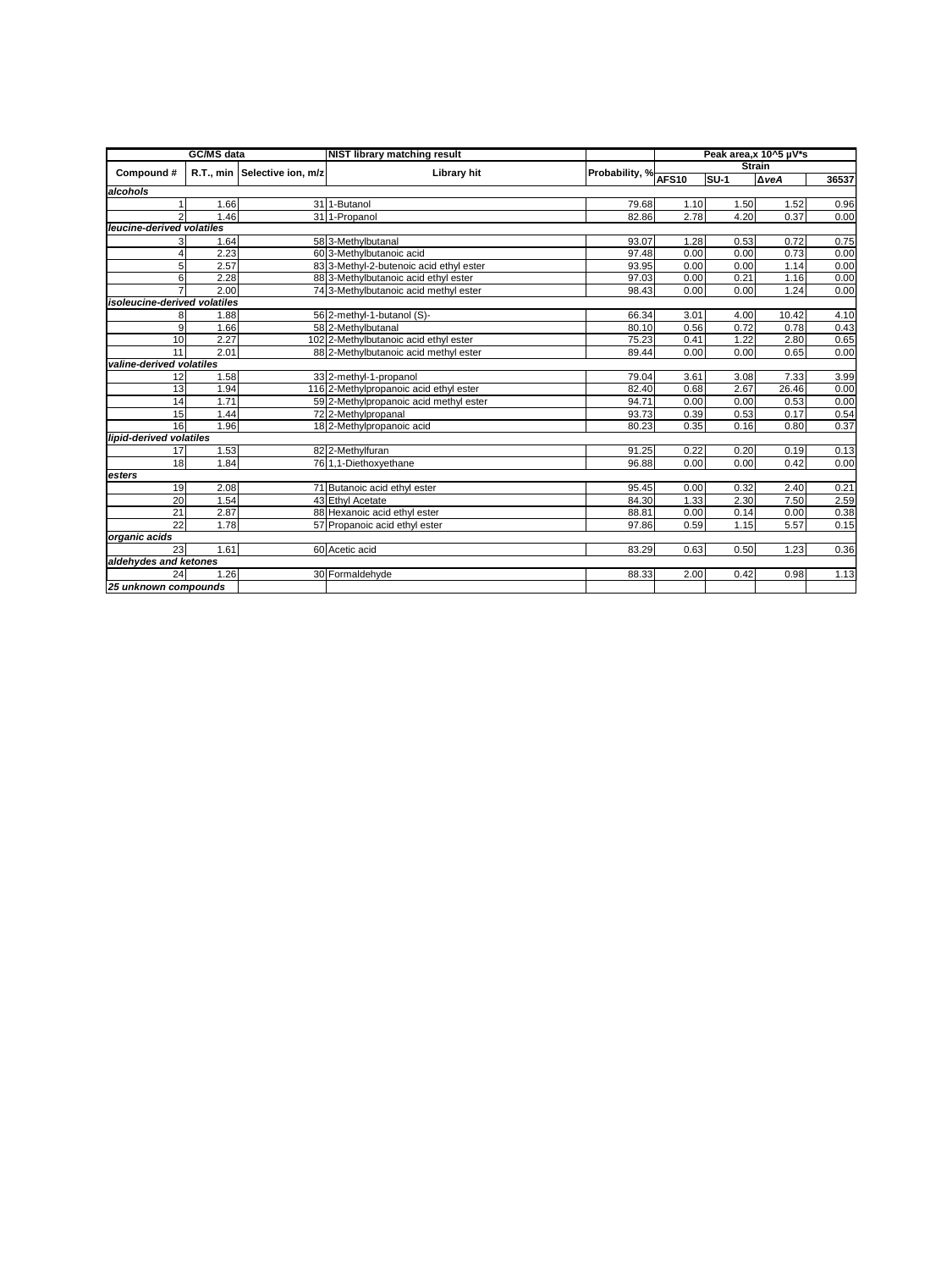

Supplement: Additional file 2 — Figure S2 - SPME-GC/MS headspace gas analysis of selected volatile compounds produced by aspergilli grown in YES medium in the dark for 72 h. Conidiospores were inoculated into 100 ml of liquid YES medium at 104/ml and the cultures were grown at 30°C, with shaking at 150 rpm, in the dark for 72 h. Each culture was grown in two individual flasks. Each experiment was conducted in triplicate. The results are presented as an average of six measurements of relative peak area × 104 + S.E. R.T., retention time, sec. [file 1471-2091-11-33-S2.PPT]

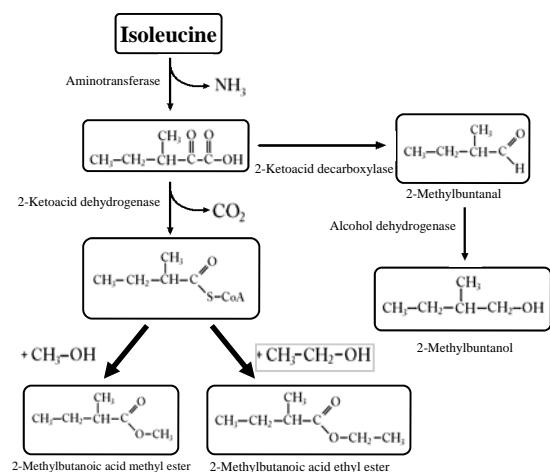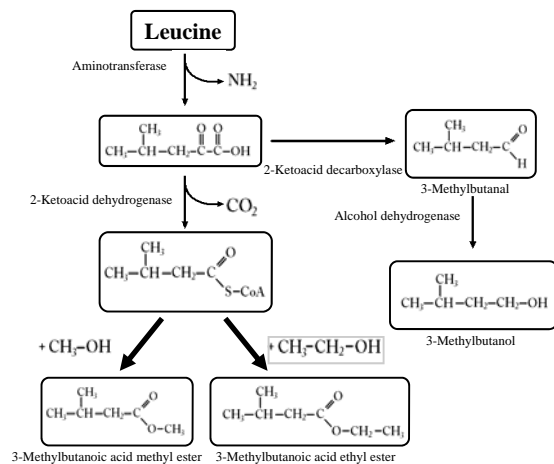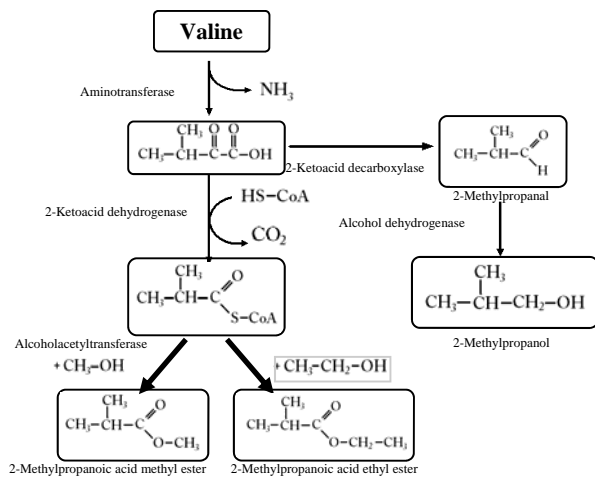

Supplement: Additional file 3 — Figure S3 - Production of fungal volatiles through pathways of branched chain amino acid catabolism. 2-ketoacids, the main intermediates, are formed through enzymatic transamination of branched chain amino acids; they can also be synthesized de novo. 2-keto acid decarboxylase leads to formation of the corresponding alcohols. 2-ketoacid dehydrogenase leads to formation of the corresponding CoA derivatives and, subsequently to methyl and ethyl esters. [file 1471-2091-11-33-S3.PDF]

## Slide 1
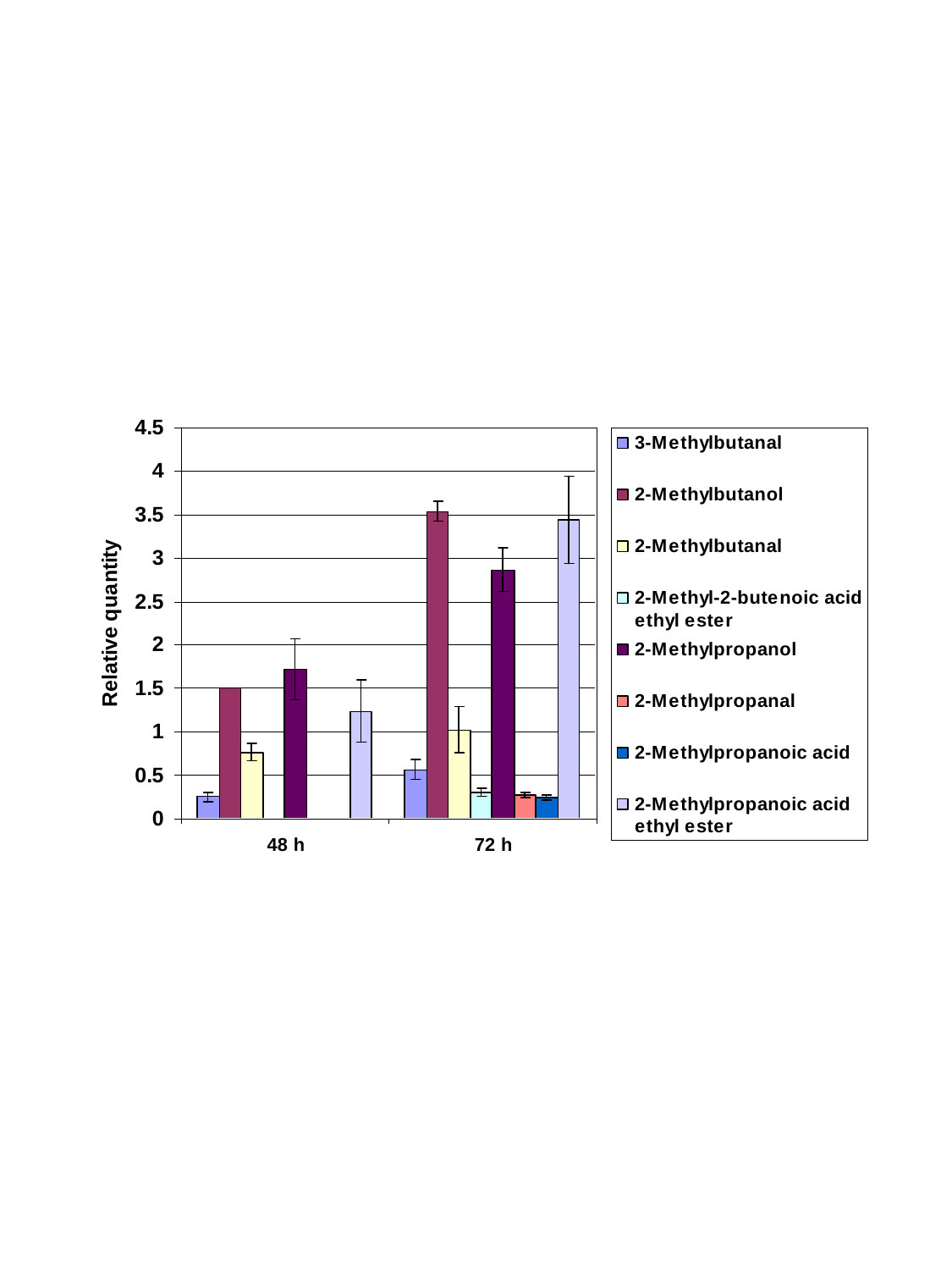

Supplement: Additional file 4 — Figure S4 - Branched chain amino acid-derived volatiles generated by SU-1 grown for 48 h and 72 h in light. Conidiospores were inoculated into 100 ml of liquid YES medium at 104/ml and the cultures were grown at 30°C, with shaking at150 rpm, in the light for 48 h and 72 h. Volatiles were analyzed as described in Methods. [file 1471-2091-11-33-S4.PPT]
