# Supplementary material for: Volatile profiling reveals intracellular metabolic changes in Aspergillus parasiticus: veA regulates branched chain amino acid and ethanol metabolism
Source: BMC Biochem. 2010 Aug 24;11:33. doi: 10.1186/1471-2091-11-33 (PMC2939540; doi:10.1186/1471-2091-11-33)
Supplement: Additional file 5 — Figure S5 - Branched chain amino acid-derived esters detected in A. parasiticus strains. *, esters unique to ΔveA. [file 1471-2091-11-33-S5.PDF]

Formula	Ester, systematic name	Amino acid
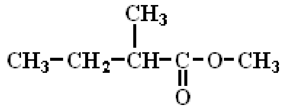	*2-Methylbutanoic acid methyl ester (gooseberry butyrate)	Ileu
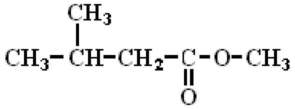	*3-Methylbutanoic acid methyl ester	Leu
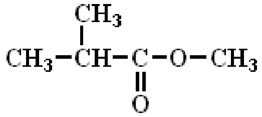	*2-Methylpropanoic acid methyl ester	Val
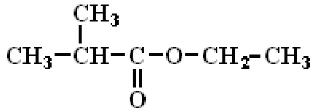	2-Methylpropanoic acid ethyl ester	Val
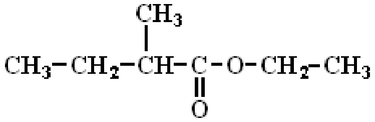	2-Methylbutanoic acid ethyl ester	Ileu
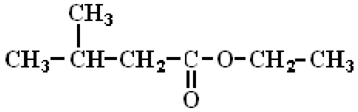	3-Methylbutanoic acid ethyl ester	Leu
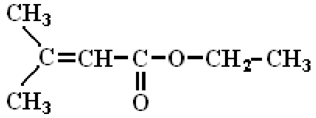	*3-Methyl-2-butenoic acid ethyl ester	Leu
