# Supplementary material for: Volatile profiling reveals intracellular metabolic changes in Aspergillus parasiticus: veA regulates branched chain amino acid and ethanol metabolism
Source: BMC Biochem. 2010 Aug 24;11:33. doi: 10.1186/1471-2091-11-33 (PMC2939540; doi:10.1186/1471-2091-11-33)
Supplement: Additional file 6 — Figure S6 - Amino acid sequence alignment of the putative A. flavus branched chain amino acid aminotransferases AFLA_113800 and AFLA_044190 with yeast BAT1 and BAT2. Amino acid sequences were aligned using Clustal multiple sequence alignment program. AFLA_113800 exhibits 61% identity to BAT1 and 60% identity to BAT2. AFLA_044190 exhibits 43% identity to BAT1 and 44% identity to BAT2. The highlighted lysine residue represents the active site of the protein in E.coli. An asterisk was added below the sequences at conserved amino acid. [file 1471-2091-11-33-S6.PDF]

```
BAT1           --MLQRHSLKLGKFSIRT----------------------LATGAPLDASKLKITRNPNP  36
BAT2           -----------------------------------------MTLAPLDASKVKITTTQHA  19
AFLA_113800    MNSLRQFTRRKALQSLAVPPSQGFSTIRLWQRHFNSTPAVASRLAGIDPSKLTVTKTSSP  60
AFLA_044190    ------------------------------------MGSVAHSVAELDASVVKITRSTNL  24
                                                           *  * *    *

BAT1           S-KPRPNEELVFGQTF-TDHMLTIPWSAKEGWGTPHIKPYGNLSLDPSACVFHYAFELFE  94
BAT2           S-KPKPNSELVFGKSF-TDHMLTAEWTAEKGWGTPEIKPYQNLSLDPSAVVFHYAFELFE  77
AFLA_113800    K-ELTPAKDLVFGKTF-TDHMLAIEWSASNGWDAPRIVPYQNLSLDPSACVFHYAFECFE  118
AFLA_044190    RPVPEPGSPEELSHSYCTDHMVTARWTVANGWETPEVKPFENLSIPPTASCLHYATECFE  84
                    *           ****    *    **  *   *  ***  * *   *** * **

BAT1           GLKAYRTPQNTITMFRPDKNMARMNKSAARICLPTFESEELIKLTGKLIEQD-KHLVPQG  153
BAT2           GMKAYRTVDNKITMFRPDMNMKRMNKSAQRICLPTFDPEELITLIGKLIQQD-KCLVPEG  136
AFLA_113800    GMKAYKDNNGQIRLFRPDKNMERLNKSSSRIALPTVDGEALTQLVGELVKLD-SRFIPSA  177
AFLA_044190    GMKVYRGYDGKLRLFRPDCNGERLLSSAQRASLPSFRYEELKVLIAKLMQIDGPRWLPKD  144
               * * *         **** *  *   *  *  **    * *  *   *   *     *

BAT1           N-GYSLYIRPTMIGTSKGLGVGTPSEALLYVITSPVG-------PYYKTGFKAVRLEATD  205
BAT2           K-GYSLYIRPTLIGTTAGLGVSTPDRALLYVICCPVG-------PYYKTGFKAVRLEATD  188
AFLA_113800    R-GYSLYLRPTMIGTQSTLGVGPPGSALLFVIASPVG-------PYYPTGFKAISLEATD  229
AFLA_044190    QPGRFLYLRPTMIGSGPHLGVQTPKEALLFIIAVPWPDPSKLKKPEEGTKPGLKLLASTP  204
                 *  ** *** **    ***  *  ***  *  *         *   *      *  *

BAT1           YATRAWPGGVGDKKLGANYAPCILPQLQAAKRGYQQNLWLFGPEKNITEVGTMNVFFVFL  265
BAT2           YATRAWPGGCGDKKLGANYAPCVLPQLQAASRGYQQNLWLFGPNNNITEVGTMNAFFVFK  248
AFLA_113800    YAVRAWPGGVGDKKLGANYAPCIVPQLSAASRGFQQNLWLFGEEEYVTEVGTMNLFIALK  289
AFLA_044190    DTIRAWPGGFGYAKLGANYGPSLVAHGKAQAIGFDQILWLFGQDRQVTEAGASNFFIVWE  264
                  ****** *  ****** *       *   *  * *****     ** *  * *
```

```
BAT1           NKVTGKKELVTAPLDG-TILEGVTRDSVLTLARDKLDPQEWDIN-----ERYYTITEVAT  319
BAT2           DSKTGKKELVTAPLDG-TILEGVTRDSILNLAKERLEPSEWTIS-----ERYFTIGEVTE  302
AFLA_113800    NKETGQKELVTAPLDG-TILEGVTRDSVLGLARERLAPNGWTVS-----ERKIRMSEVAE  343
AFLA_044190    NKETGKIELVTAPLENQLILPGVTRRSVLQLARTELSKPTGSLAPVEVVERDFTISEVEQ  324
                  **  *******    ** **** * * **   *             **     **

BAT1           RAKQGELLEAFGSGTAAVVSPIKEIGWNNEDIHVPLLPGEQCGALTKQVAQWIADIQYGR  379
BAT2           RSKNGELLEAFGSGTAAIVSPIKEIGWKGEQINIPLLPGEQTGPLAKEVAQWINGIQYGE  362
AFLA_113800    AADEGRLIEVFGSGTAAIVSPVRNISYKGKMVDCGLKEDEEAGEIASQMKNWIEGIQYGD  403
AFLA_044190    AWKEGRIIEAFVCGTAFFVTPVKLIRNGDVDMDM-LEAGAARGGYAVQIKSWLEAIMYGK  383
                   *   * *  ***  * *   *          *      *        *   * **

BAT1           --VNYGNWSKTVADLN--  393
BAT2           --TEHGNWSRVVTDLN--  376
AFLA_113800    --EDH-KWSYVL------  412
AFLA_044190    DGKENDEWSYIIEGESEK  401
                      **
```
