# Supplementary material for: Volatile profiling reveals intracellular metabolic changes in Aspergillus parasiticus: veA regulates branched chain amino acid and ethanol metabolism
Source: BMC Biochem. 2010 Aug 24;11:33. doi: 10.1186/1471-2091-11-33 (PMC2939540; doi:10.1186/1471-2091-11-33)
Supplement: Additional file 7 — Figure S7 - Amino acid sequence alignment of the putative A. flavus alcohol dehydrogenase, AFLA_048690, with the yeast alcohol dehydrogenase, ADH1. Amino acid sequences were aligned using Clustal multiple sequence alignment program. AFLA_048690 exhibits 57% identity to yeast ADH1. An asterisk was added below the sequences at conserved amino acid. [file 1471-2091-11-33-S7.PDF]

```
ADH1            MSIPETQKGVIFYESHGKLEHKDIPVPKPKANELLINVKYSGVCHTDLHAWHGDWPLPVK  60
AFLA_048690     MSIPEMQWAQVAEQKGGPLIYKQIPVPKPGPDEILVKVRYSGVCHTDLHALKGDWPLPVK  60
                ***** *         * *  * ******   * *  * ***********  ********

ADH1            LPLVGGHEGAGVVVGMGENVKGWKIGDYAGIKWLNGSCMACEYCELGNESNCPHADLSGY  120
AFLA_048690     MPLVGGHEGAGVVVARGDLVTEFEIGDHAGLKWLNGSCLACEFCKQADEPLCPNASLSGY  120
                 *************  *  *    *** ** ******* *** *    *  ** * ****

ADH1            THDGSFQQYATADAVQAAHIPQGTDLAQVAPILCAGITVYKALKSANLMAGHWVAISGAA  180
AFLA_048690     TVDGTFQQYAIGKATHASKLPKNVPLDAVAPVLCAGITVYKGLKESGVRPGQTVAIVGAG  180
                * ** *****   *  *   *    *  *** ********* **      *  *** **

ADH1            GGLGSLAVQYAKAMGYRVLGIDGGEGKEELFRSIGGEVFIDFTKEKDIVGAVLKATDG--  238
AFLA_048690     GGLGSLALQYAKAMGIRVVAIDGGEEKQAMCEQLGAEAYVDFTKTQDLVADVKAATPEGL  240
                ******* ******* **  ***** *       * *   ****  * *  *  **

ADH1            GAHGVINVSVSEAAIEASTRYVRANGTTVLVGMPAGAKCCSDVFNQVVKSISIVGSYVGN  298
AFLA_048690     GAHAVILLAVAEKPFQQAAEYVRSRGTVVAIGLPAGAFLRAPVFNTVVRMINIKGSYVGN  300
                *** **   * *        ***  ** *  * ****     *** **  * * ******

ADH1            RADTREALDFFARGLVKSPIKVVGLSTLPEIYEKMEKGQIVGRYVVDTSK  348
AFLA_048690     RQDGVEAVDFFARGLIKAPFKTAPLQDLPKIFELMEQGKIAGRYVLEIPE  350
                * *  ** ******* * * *   *  ** * * ** * * ****
```
